# Cooperative Dynamic Game-Based Optimal Power Control in Wireless Sensor Network Powered by RF Energy

**DOI:** 10.3390/s18072393

**Published:** 2018-07-23

**Authors:** Manxi Wang, Haitao Xu, Xianwei Zhou

**Affiliations:** 1 State Key Laboratory of Complex Electromagnetic Environment Effects on Electronics and Information System, Luoyang 471003, China; cemee@vip.163.com; 2 School of Computer and Communication Engineering, University of Science and Technology Beijing, Beijing 100083, China; xwzhouli@sina.com

**Keywords:** cooperative dynamic game, power control, wireless sensor network, RF energy transfer

## Abstract

This paper focuses on optimal power control in wireless sensor networks powered by RF energy, under the simultaneous wireless information and power transfer (SWIFT) protocol, where the information and power can be transmitted at the same time. We aim to maximize the utility for each sensor through the optimal power control, considering the influences of both the *SINR* and the harvested energy. The utility maximization problem is formulated as a cooperative dynamic game of a given time duration. All the sensors cooperate together to control their transmission power to maximize the utility and agree to act cooperatively so that a team optimum can be achieved. As a result, a feedback Nash equilibrium solution for each sensor is given based on the dynamic programming theory. Simulation results verify the effectiveness of the proposed approach, by comparing the grand coalition solutions with the non-cooperative solutions.

## 1. Introduction

As an important component of wireless networks, wireless sensor networks have drawn lots of academic and industrial research interest for a long time, as wireless sensor networks with sensing, computation, and communication capabilities can work autonomously [1]. More and more sensor nodes are arranged to constitute the wireless sensor networks to realize the concept of the Internet of Things (IoT) [2].

In a traditional wireless sensor network, the wireless sensors should constantly transmit the collected information to the access point. Meanwhile, they either have limited battery energy, or are powered by grid energy sources [3,4]. For the wireless sensors with limited battery power, their working time is restricted by their limited energy. For the wireless sensors powered by grid energy sources, they will be restricted to a fixed area. Radio frequency (RF)-based wireless energy transfer can be introduced to solve the limited energy and non-stationary power supply problems [5]. Through RF-based wireless energy transfer, the wireless sensors can replenish their energy from various energy sources [6]. Applying the wireless energy transfer into wireless sensor networks, can enhance the life cycle of the sensor nodes, and improve the network performance [7].

In wireless sensor networks, even when the wireless sensors are powered by the RF energy, the energy consumption problem is still a severe problem [8], because of the increasing demand for computing and communication tasks [9]. Therefore, how to control the energy consumption in wireless sensor networks, especially how to control the sensors’ power level for information transmission, is still a continuing scientific problem that needs to be solved [10]. Lots of works have been done in the power control problems in the wireless sensor networks powered by RF energy [11,12,13,14]. In [11], a wireless powered sensor network with minimal power requirements is designed. The energy consumption of the circuit and information processing are considered in the proposed model. The effect of the number of sensors are also taken into account. In [12], a proper MAC protocol is designed to solve the problem of the tradeoff between the RF energy transfer and data communication. Meanwhile, a corresponding Markov chain m and steady-state probabilities are derived to fulfill the performance analysis. In [13], a fuzzy power allocation and rate adaptation model is proposed. Throughput and energy performance are analyzed for the proposed scheme. Kwan et al. [14] designed an optimal protocol for data transmission considering RF energy harvesting. A multi-source selection and timing allocation algorithm is proposed and a closed-form mathematical solution is given.

Nevertheless, none of the above works to achieve optimal power control in wireless sensor networks powered by RF energy consider the dynamic characteristics of the battery energy, and do not consider the optimization in given a time period. In this paper, we try to use dynamic game theory by considering the dynamic characteristics of the battery and try to solve the optimal power control problem in a time period. The dynamic game theory, also called differential game theory, was firstly proposed by Isaace [15], is one of the most practical and complex branches of game theory and can be used to solve a class of resource allocation problems, under which the evolution of the state is described by a differential equation and the players act throughout a time interval [16]. A non-cooperative dynamic game-based power control model has been proposed in [17], to solve the power control problem in wireless powered sensor network using feedback control. In this paper, based on the cooperative dynamic game, we pay attention to the power control problem in wireless sensor networks which are powered by RF energy, to maximize the utility based on the *SINR* requirements and the energy state. We aim at finding an optimal strategy for the sensors mapping the power level to the *SINR* requirements and the energy state. The game solutions are gotten in the condition of grand coalition and non-cooperative feedback Nash equilibrium. The Shapely theorem is used to achieve fairly allocation. The main contributions of this work are summarized as follows:

- Firstly, we formulate the system model of the wireless sensor network powered by RF energy, which consists of one access point and *N* sensor nodes, where the sensor nodes can harvest energy and transmit information simultaneously.
- Secondly, a dynamic game model is proposed to formulate the power control problem in the proposed network. The energy variations are considered as the system state, and the objective function is composed by the *SINR* and energy requirements.
- Finally, two kinds of analyses are given, which are the grand coalition solutions and non-cooperative solutions for the sensors.

The remainder of the paper is organized as follows: Section 2 introduces the system model of wireless sensor networks powered RF energy and the power control problem in a dynamic game. Section 3 provides the grand coalition solutions and the feedback Nash equilibrium solutions for each wireless sensor. Numerical simulations are given in Section 4. Finally, we conclude the work in Section 5.

## 2. System Model and Problem Formulation

### 2.1. System Model

**Figure 1:**
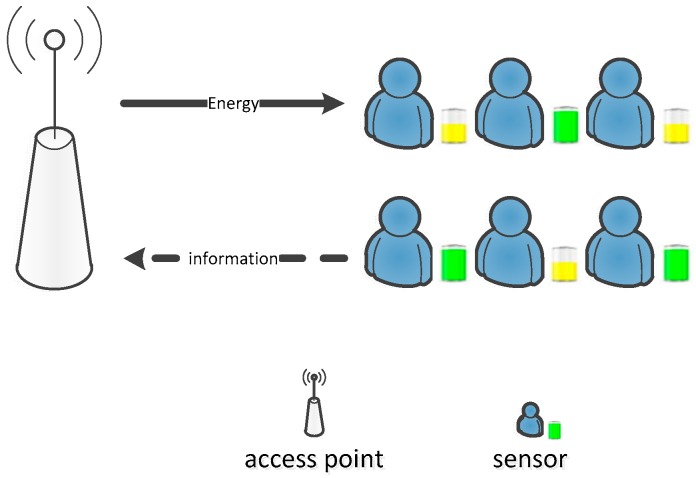
Wireless sensor network powered by RF energy.

**Figure 2:**
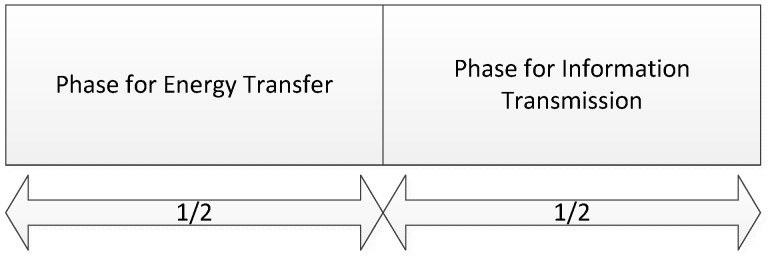
Time switching (TS) protocol.

Consider a wireless sensor network powered by RF energy with one access point (AP) and *N* sensors, where the sensors are equipped with rechargeable batteries and can obtain energy from the AP based on RF energy transfer, as shown in Figure 1. Located at an appropriate place, the AP has abilities to transfer energy to all sensors, and can work as a data gathering point to collect and transmit information for all sensors. The AP is connected to a constant power supply, and the broadcast energy over RF signals is assumed to be fixed on a stable level for all sensors. In this paper, we assume that the AP can be serve as a sink node [4] for information transmission, which operates on 2.4 GHz. Then all the sensors can transmit information to the AP directly. For wireless energy transfer, the AP can utilize the spectrum at 350 MHz to 3 GHz to carry RF energy to the sensor nodes [18]. As the sensors are equipped with limited rechargeable batteries, they need to harvest energy from the AP and use the harvested energy to transmit information. The simultaneous wireless information and power transfer (SWIFT) is applied. Both AP and sensors are equipped with two antennas, for wireless energy transfer (WET) and wireless information transmission (WIT) individually. Meanwhile, we assume that the wireless energy transfer and the information transmission operate over orthogonal frequency bands with identical bandwidth, and thus the sensor nodes can harvest energy and transmit information at the same time. Wireless energy and information transmission are operated at the same frequency, based on the “harvest-then-transmit” protocol [19], as shown in Figure 2. To simplify the analysis, the time durations for energy transfer and information transmission are assumed to be the same in this paper.

As the sensors have limited energy, so it is essential to control the information transmission power for all the sensors, even they are powered by the RF energy. Then the target of this paper is to get the optimal uplink information transmission power in the wireless sensor networks. We model the power control problem as a cooperative dynamic game, where all sensors try to cooperate together. Meanwhile, we will consider the requirements of signal-to-interference-plus-noise ratio (*SINR*) and residual energy after information transmission for model construction.

### 2.2. Energy State

During the process of downlink WET, the sensors will harvest energy from the AP, and prepare enough energy for the uplink information transmission. The amount of harvested energy at sensor *i* is denoted by *q_i_* and can be expressed as follows:

(1) $$q_{i}=\eta G_{i}q,$$

where *q* is the transferred energy from the AP. As the transferred energy from the AP is a broadcasting energy, it is assumed to be the same for all sensors. *η* is the energy conversion efficiency. Let *η* = 1 for simplification. *G_i_* denotes the channel power gain between the AP and sensor *i*. As noise can be ignored for energy transfer, we assume there are no harvested energy from noise.

Sensors equipped with rechargeable batteries can use the harvested energy for information transmission. In this paper, we assume all the sensors can transmit information and harvest energy at the same time. Let *x* denote the batteries energy of all the sensors, which can be considered as the state of the system. Assuming that the batteries energy being decreased by the uplink power consumed by information transmission and being increased by the harvested energy from AP in a linear relationship. Let *p_i_* denote the information transmission power of sensor i, then the evaluation of *x* can be expressed by the following differential equation:

(2) $$dx/dt=\sum_{i=1}^{N}\left(q_{i}-p_{i}\right)+\delta x=\sum_{i=1}^{N}\left(\eta G_{i}q-p_{i}\right)+\delta x,$$

where *δ* is a time-varying parameter of energy, and can be expressed as *δ = ηq*/*E*, with *E* is the maximum battery capacity of the sensors.

During the process of uplink WIT, the sensors will control their information transmission power based on the *SINR* requirements. Because the wireless sensors can share the same spectrum for the uplink WIT and the downlink WET, the interference to sensor *i* should mainly come from the WET of AP. Assuming *n*_0_ is the power spectral density of the additive white Gaussian noise, then the *SINR* for sensor *i* can be expressed as:

(3) $$\gamma_{i}=\frac{p_{i}}{n_{0}+q}=\frac{1}{\alpha_{i}}p_{i}.$$

In (3), as *q* is a constant power for all sensors, we can re-write the above formula with *αi* and $\alpha_{i}=n_{0}+q$. Then we have $p_{i}=\alpha_{i}\gamma_{i}$, and Equation (2) can be reformulated as follows:

(4) $$dx/dt=\sum_{i=1}^{N}\left(\eta G_{i}q-p_{i}\right)+\delta x=\sum_{i=1}^{N}\left(\eta G_{i}q-\alpha_{i}\gamma_{i}\right)+\delta x.$$

### 2.3. Problem Formulation

Based on (3), we can see that the *SINR* is in direct proportion to the uplink information transmission power level. For each sensor, it expects to increase the uplink WIT power to achieve higher *SINR*, which means the sensors can earn more “profit” for higher *SINR* when increasing the uplink WIT power level. Assuming there is a *SINR* threshold for each sensor and is denoted by $\bar{\gamma_{i}}$, then the profit for having a higher *SINR* can be expressed as:

(5) $${\mathrm{Pr}}_{SINR}^{i}=\frac{1}{2}\beta_{i}{\left(\gamma_{i}-\bar{\gamma_{i}}\right)}^{2}.$$

Besides the higher *SINR* profit, profit of battery energy is also considered in our model. We define the profit of battery energy is a linear form of the battery energy and can be expressed as:

(6) $${\mathrm{Pr}}_{battery}^{i}=\pi_{i}x,$$

where all sensors’ contributions for battery profit are denote by the contributions parameter $\pi_{i}$.

To maximize the *SINR* and the final energy among all sensors, the utility of each sensor is defined as the combination of achievable *SINR* and energy level, which is given:

(7) $$\begin{array}{ll} \max U\left(x,t\right) & =\int_{0}^{\infty }{\mathrm{Pr}}_{i}ds=\int_{0}^{\infty }\left({\mathrm{Pr}}_{SINR}^{i}+{\mathrm{Pr}}_{battery}^{i}\right)ds=\int_{0}^{\infty }\left(\frac{1}{2}\beta_{i}{\left(\gamma_{i}-\bar{\gamma_{i}}\right)}^{2}+\pi_{i}x\right)ds\\ & =\int_{0}^{\infty }\left(\frac{1}{2}\beta_{i}{\left(\frac{1}{\alpha_{i}}p_{i}-\bar{\gamma_{i}}\right)}^{2}+\pi_{i}x\right)ds \end{array},$$

s.t. (4).

Now, we formulate the optimal power control for all sensors as a cooperative dynamic game, as follows:

- Players: All wireless sensors.
- Strategy space: All wireless sensors can cooperatively choose their information transmit power to maximize the utility given in (7).
- State: The battery energy state is denoted by vector *x*, where the state is controlled by the dynamic constraint in Equation (4).
- Objective function: All of the wireless sensors act to maximize their utility.

## 3. Solutions and Analysis

In this section, we will analyse the solutions to the game problem given in (7) based on the dynamic optimization programming technique, which was introduced by Bellman. We try to get the feedback Nash equilibrium solutions for all the sensors. We consider the case when all the sensors cooperate together to control their transmission power to maximize the profit, and agree to act so that a team optimum could be achieved. In the cooperative dynamic game, the group rationality and individual rationality should be satisfied at any instant of interval time.

**Lemma** **1.** *For the optimization Equations (7), an n-tuple of strategies*$\left\{p_{i}^{*}\left(t,x\right),for\;i\in N\right\}$*constitutes a feedback Nash equilibrium solution if there exists a functional*$V^{i}\left(t,x\right)$*, defined on the time interval*[0,T]*and satisfying the following relations for each*i∈N*[20,21]*:

(8) $$V^{i}\left(t,x\right)==\int_{0}^{\infty }\left(\frac{1}{2}\beta_{i}{\left(\frac{1}{\alpha_{i}}p_{i}{}^{*}-\bar{\gamma_{i}}\right)}^{2}+\pi_{i}x\right)ds\geq \int_{0}^{\infty }\left(\frac{1}{2}\beta_{i}{\left(\frac{1}{\alpha_{i}}p_{i}-\bar{\gamma_{i}}\right)}^{2}+\pi_{i}x\right)ds.$$

Then, we will give the process for obtaining the cooperative solutions as follows.

### 3.1. Computation of Optimal Cost of Grand Coalition

For each sensor, its target is to maximize the profit given in (7). In order to get the optimal solution to the game (7), firstly we should define the value function based on the dynamic optimization programming. The value function W(N,x,t) must satisfy the Bellman equation:

(9) $$rW\left(N,x,t\right)=\underset{\gamma_{i}}{\max}\left\{\sum_{i=1}^{N}\left[\frac{1}{2}\beta_{i}{\left(\gamma_{i}-\bar{\gamma_{i}}\right)}^{2}+\pi_{i}x\right]+W_{x}\left(N,x,t\right)\left[-\sum_{i=1}^{N}\alpha_{i}\gamma_{i}+\delta x+\sum_{i=1}^{N}\left(\eta G_{i}q\right)\right]\right\}.$$

Performing the indicated minimization in (9) yields:

(10) $$\left\{ \begin{array}{l} \gamma_{i}{}^{N}=\bar{\gamma_{i}}+\frac{\alpha_{i}}{\beta_{i}}W_{x}\left(N,x,t\right)\\ p_{i}^{N}=\alpha_{i}\bar{\gamma_{i}}+\frac{\alpha_{i}{}^{2}}{\beta_{i}}W_{x}\left(N,x,t\right) \end{array}\right..$$

Substituting $\gamma_{i}^{N}$ upon into (9) and solving, we can yield the value function as follows:

(11) $$W\left(N,x,t\right)=\frac{\sum_{i=1}^{N}\pi_{i}}{r\left(r-\delta \right)}\left\{\sum_{i=1}^{N}\left(-\alpha_{i}\bar{\gamma_{i}}-\frac{1}{2}\frac{\alpha_{i}^{2}}{\beta_{i}}\frac{\sum_{i=1}^{N}\pi_{i}}{r-\delta }\right)+rx+\sum_{i=1}^{N}\left(\eta G_{i}q\right)\right\}.$$

Let $\bar{\pi }=\sum_{i=1}^{N}\pi_{i}$, then we have:

(12) $$W\left(N,x,t\right)=\frac{\bar{\pi }}{r\left(r-\delta \right)}\left\{\sum_{i=1}^{N}\left(-\alpha_{i}\bar{\gamma_{i}}-\frac{1}{2}\frac{\alpha_{i}^{2}}{\beta_{i}}\frac{\bar{\pi }}{r-\delta }\right)+rx+\sum_{i=1}^{N}\left(\eta G_{i}q\right)\right\}.$$

Based on (12), the optimal *SINR* and transmit power of sensor *i* can be given by:

(13) $$\left\{ \begin{array}{l} \gamma_{i}{}^{N}=\bar{\gamma_{i}}+\frac{\alpha_{i}\bar{\pi }}{\beta_{i}\left(r-\delta \right)}\\ p_{i}^{N}=\alpha_{i}\bar{\gamma_{i}}+\frac{\alpha_{i}{}^{2}\bar{\pi }}{\beta_{i}\left(r-\delta \right)} \end{array}\right..$$

and we can get the optimal trajectory of battery energy as follows:

(14) $$x^{N}\left(t\right)=\exp\left(\delta t\right)x\left(0\right)+\frac{1}{\delta }\left\{\sum_{i=1}^{N}\left(-\alpha_{i}\gamma_{i}{}^{N}\right)\right\}\left(1-\exp\left(\delta t\right)\right).$$

Based on the above equations, we have obtained the optimal *SINR* and transmission power for each sensor and the maximized utility in grand coalition. The battery energy of all the sensors in grand coalition condition, which are the state of the wireless powered sensor networks, can also be obtained based on (14). From (14), we can find that the optimal trajectory of the battery is a function of the optimal *SINR* for each sensor, with an initial energy level x(0). It can be seen that the optimal variation of the energy is an exponential function, which fits the physical meaning of the battery. Through (14), we can obtain the optimal variation of the energy state in the proposed wireless sensor networks, under grand coalition condition.

### 3.2. Computation of Feedback Nash Equilibrium

To solve the feedback Nash Equilibrium for the game (7), the following Bellman equation should be satisfied:

(15) $$rV^{i}\left(x\right)=\underset{\gamma_{i}}{\max}\left\{\frac{1}{2}\beta_{i}{\left(\gamma_{i}-\bar{\gamma_{i}}\right)}^{2}+\pi_{i}x+V_{x}^{i}\left(x\right)\left[-\alpha_{i}\gamma_{i}-\sum_{j=1,j\neq i}^{N}\left(\alpha_{j}\gamma_{j}\right)+\delta x+\sum_{i=1}^{N}\left(\eta G_{i}q\right)\right]\right\},\;\mathrm{for}\;i\in N.$$

Similar to Section 3.2, we can get the indicated minimization of (15) as follows:

(16) $$\left\{ \begin{array}{l} \gamma_{i}=\bar{\gamma_{i}}+\frac{\alpha_{i}}{\beta_{i}}V_{x}^{i}\left(x\right)\\ p_{i}=\alpha_{i}\bar{\gamma_{i}}+\frac{\alpha_{i}{}^{2}}{\beta_{i}}V_{x}^{i}\left(x\right) \end{array}\right..$$

Substituting (16) into the Bellman Equation (15) and solving, we can yield the following results:

(17) $$V_{x}^{i}\left(x\right)=\frac{\pi_{i}}{r-\delta },$$

(18) $$V^{i}\left(x\right)=\frac{\pi_{i}}{r\left(r-\delta \right)}\left\{\left[\sum_{j=1}^{N}\left(-\alpha_{j}\bar{\gamma_{j}}-\frac{\alpha_{j}^{2}}{2\beta_{j}}\frac{\pi_{j}}{r-\delta }\right)-\sum_{j=1,j\neq i}^{N}\left(\frac{\alpha_{j}^{2}}{2\beta_{j}}\frac{\pi_{j}}{r-\delta }\right)\right]+rx+\sum_{i=1}^{N}\left(\eta G_{i}q\right)\right\}.$$

and the feedback Nash equilibrium level can then be obtained as:

(19) $$\left\{ \begin{array}{l} \gamma_{i}=\bar{\gamma_{i}}+\frac{\alpha_{i}}{\beta_{i}}\frac{\pi_{i}}{r-\delta }\\ p_{i}=\alpha_{i}\bar{\gamma_{i}}+\frac{\alpha_{i}{}^{2}}{\beta_{i}}\frac{\pi_{i}}{r-\delta } \end{array}\right..$$

The difference between Nash equilibrium obtained in (19) and those obtained for the grand coalition in (13) is that player takes into account the sum of all coalition members and not only his own one.

### 3.3. Computation of Optimal Cost for Intermediate Coalitions 

The value function W(K,x,t) for the players in coalition K(|K|<N) must satisfy the following Bellman equation:

(20) $$\begin{array}{l} rW\left(K,x,t\right)=\underset{\gamma_{i},i\in K}{\max}\{\sum_{i\in K}\left[\frac{1}{2}\beta_{i}{\left(\gamma_{i}-\bar{\gamma_{i}}\right)}^{2}+\pi_{i}x\right]\\ +W_{x}\left(K,x,t\right)\left[-\sum_{i\in K}\alpha_{i}\gamma_{i}-\sum_{j\in NK}\alpha_{j}\gamma_{j}^{*}+\delta x+\sum_{i=1}^{N}\left(\eta G_{i}q\right)\right]\} \end{array}$$

Performing the indicated minimization to (20) yields:

(21) $$\left\{ \begin{array}{l} \gamma_{i}^{K}=\bar{\gamma_{i}}+\frac{\alpha_{i}}{\beta_{i}}W_{x}\left(K,x,t\right)\\ p_{i}=\alpha_{i}\bar{\gamma_{i}}+\frac{\alpha_{i}{}^{2}}{\beta_{i}}W_{x}\left(K,x,t\right) \end{array}\right.,$$

(22) $$W_{x}\left(K,x,t\right)=\frac{\sum_{i\in K}\pi_{i}}{r-\delta }.$$

Substituting (21) and (22) into the Equation (20) and solving yield:

(23) $$W\left(K,x,t\right)=\frac{\sum_{i\in K}\pi_{i}}{r\left(r-\delta \right)}\left\{\sum_{i\in K}\left(-\alpha_{i}\bar{\gamma_{i}}-\frac{\alpha_{i}{}^{2}}{2\beta_{i}}\frac{\sum_{i\in K}\pi_{i}}{r-\delta }\right)-\sum_{j\in NK}\alpha_{j}\left(\bar{\gamma_{j}}+\frac{\alpha_{j}\pi_{j}}{\beta_{j}\left(r-\delta \right)}\right)+rx+\sum_{i=1}^{N}\left(\eta G_{i}q\right)\right\}.$$

### 3.4. Definition of the Characteristic Function and Computation of the Shapley Value

The values of the characteristic function are given by:

(24) $$v\left(\left\{i\right\};x,t\right)=V^{i}\left(x\right)=\frac{\pi_{i}}{r\left(r-\delta \right)}\left\{\left[\sum_{j=1}^{N}\left(-\alpha_{j}\bar{\gamma_{j}}-\frac{\alpha_{j}^{2}}{2\beta_{j}}\frac{\pi_{j}}{r-\delta }\right)-\sum_{j=1,j\neq i}^{N}\left(\frac{\alpha_{j}^{2}}{2\beta_{j}}\frac{\pi_{j}}{r-\delta }\right)\right]+rx+\sum_{i=1}^{N}\left(\eta G_{i}q\right)\right\},$$

(25) $$\begin{array}{l} v\left(K;x,t\right)=W\left(K,x,t\right)=\frac{\sum_{i\in K}\pi_{i}}{r\left(r-\delta \right)}\{\sum_{i\in K}\left(-\alpha_{i}\bar{\gamma_{i}}-\frac{\alpha_{i}{}^{2}}{2\beta_{i}}\frac{\sum_{i\in K}\pi_{i}}{r-\delta }\right)\\ -\sum_{j\in NK}\alpha_{j}\left(\bar{\gamma_{j}}+\frac{\alpha_{j}\pi_{j}}{\beta_{j}\left(r-\delta \right)}\right)+rx+\sum_{i=1}^{N}\left(\eta G_{i}q\right)\} \end{array}.$$

In order to be convenient for computing the Shapley value and clarifying our model, we suppose *N* = 3, then we have:

(26) $$\begin{array}{l} \phi_{i}^{v}\left(x,t\right)=\frac{\sum_{i\in K}\pi_{i}}{r\left(r-\delta \right)}\left\{\sum_{h\in K,h\neq i}\left(-\alpha_{h}\bar{\gamma_{h}}-\frac{\alpha_{h}{}^{2}}{2\beta_{h}}\frac{\sum_{i\in K}\pi_{i}}{r-\delta }\right)-\sum_{j\in NK}\alpha_{j}\left(\bar{\gamma_{j}}+\frac{\alpha_{j}\pi_{j}}{\beta_{j}\left(r-\delta \right)}\right)\right\}\\ -\frac{\pi_{i}}{r\left(r-\delta \right)}\sum_{j=1,j\neq i}^{N}\left(-\alpha_{j}\bar{\gamma_{j}}-\frac{\alpha_{j}^{2}}{\beta_{j}}\frac{\pi_{j}}{r-\delta }\right)+\frac{\sum_{i\in K}\pi_{i}-\pi_{i}}{r-\delta }x+\sum_{i=1}^{N}\left(\eta G_{i}q\right) \end{array}.$$

### 3.5. Computation of IDP Functions 

In [22], the authors defined the Imputation Distribution Procedure (IDP) being $B\left(t\right)=\left\{B_{1}\left(t\right),B_{2}\left(t\right),...,B_{N}\left(t\right)\right\}$, and for the time constant $B_{i}\left(t\right)$, it can be calculated as follows:

(27) $$B_{i}\left(t\right)=\phi_{i}^{v}\left(x_{t}^{N},t\right)-\frac{d}{dt}\phi_{i}^{v}\left(x_{t}^{N},t\right).$$

In (27), we can find that the IDP function is a function of the Shapley values. Combining the Shapley values obtained in the Section 3.4, we can get the final allocation for each sensor.

## 4. Numerical Results

In this section, we will simulate the method proposed in Section 3. Based on [10], assuming there are three wireless nodes powered by one access point. Each sensor needs to control the information transmission power to maximize the network profit. The grand coalition and feedback Nash equilibrium solutions introduced in Section 3 are simulated to get different results under different situations.

**Figure 3:**
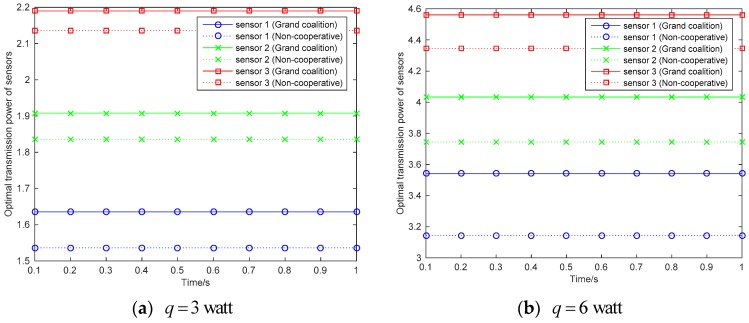
Optimal power level for information transmission of each sensor.

Figure 3 shows the optimal power level of each sensor for information transmission. In Figure 3a, the power for energy transfer is set to be 3 Watt, where it is set to be 6 Watt in Figure 3b. It can be seen that the sensors can have more energy for information transmission when they can harvest more energy form the RF energy. Two kinds of solutions are obtained for all sensors, which are grand coalition solutions and non-cooperative solutions respectively. The power level for information transmission is higher in grand coalition than the non-cooperative solutions. This represents that the power is more efficiently used in grand coalition. In other words, the grand coalition can inspire the sensors working efficiently aiming at maximize the profit.

**Figure 4:**
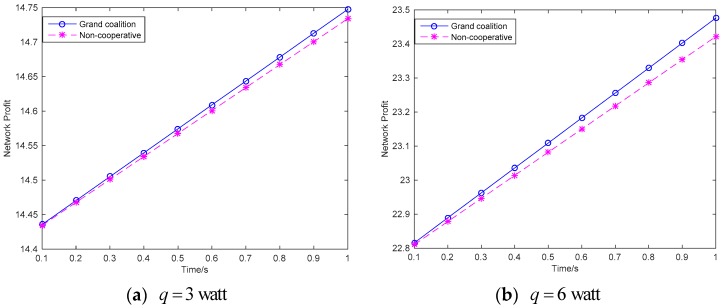
Network profit.

**Figure 5:**
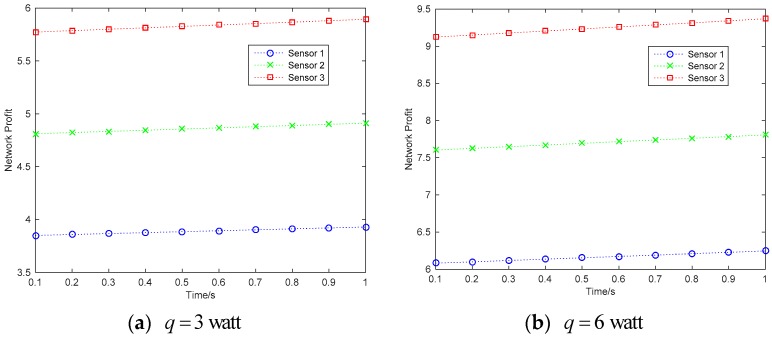
Profit of each sensor.

Figure 4 shows the maximized network profit of the wireless sensor networks. The conclusion is that the network profit is increased with the time variation. Meanwhile, the network profit is higher in grand coalition that that in non-cooperative condition. In the condition of grand coalition, based on the simulations given in Figure 3, the sensors will have more power for information transmission, then they have more willing to cooperative together to maximize the network profit. Figure 5 shows the maximized profit of each sensor under the grand coalition condition and the non-cooperative condition respectively.

**Figure 6:**
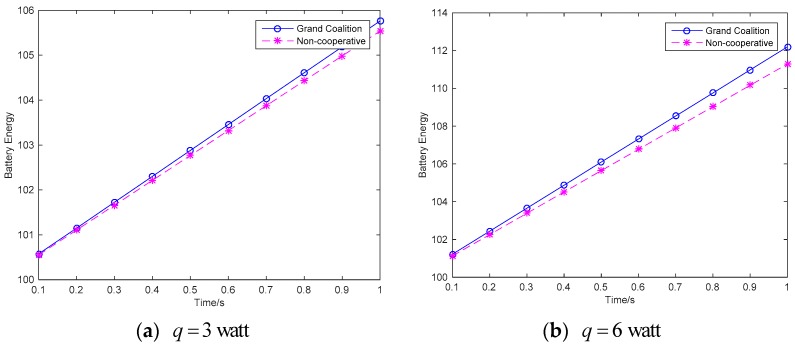
Battery energy variation.

Figure 6 shows the energy variation of the wireless sensor networks. With the energy transfer, the energy of the wireless sensor network is increased with the time variation. In the guarantee of the quality of services, each sensor will try to reserve more energy to maximize the network utility.

## 5. Conclusions

In this paper, we have proposed a cooperative dynamic game-based model that maximizes the network utility considering the *SINR* requirements and energy variations, achieved by cooperatively optimal allocation of the information transmission power. In the proposed game model, the researched wireless sensor networks are powered by the RF energy sources. The energy variations are considered as the system state of the wireless sensor networks, and the sensors can control their information transmission power based on the grand coalition solutions and the non-cooperative Nash equilibrium. Based on the simulation results, it can be seen that our proposed model can achieve optimal power control.
